# CHCHD4 regulates tumour proliferation and EMT-related phenotypes, through respiratory chain-mediated metabolism

**DOI:** 10.1186/s40170-019-0200-4

**Published:** 2019-07-16

**Authors:** Luke W. Thomas, Cinzia Esposito, Jenna M. Stephen, Ana S. H. Costa, Christian Frezza, Thomas S. Blacker, Gyorgy Szabadkai, Margaret Ashcroft

**Affiliations:** 1 Department of Medicine, University of Cambridge, Cambridge Biomedical Campus, Cambridge, CB2 0AH UK; 2 Medical Research Council Cancer Unit, University of Cambridge, Hutchison/MRC Research Centre, Cambridge Biomedical Campus, Box 197, Cambridge, CB2 0XZ UK; 3 Department of Cell and Developmental Biology, Division of Biosciences, University College London, Gower Street, London, WC1E 6BT UK; 4 Present Address: Department of Molecular Life Sciences, University of Zurich, Winterthurerstrasse 190, CH-8057 Zurich, Switzerland

**Keywords:** Coiled-coil helix coiled-coil helix domain-containing protein 4 (CHCHD4), hypoxia, HIF-1α, mitochondria, respiratory chain, disulfide relay system, complex I, tumour growth, tumour metabolism

## Abstract

**Background:**

Mitochondrial oxidative phosphorylation (OXPHOS) via the respiratory chain is required for the maintenance of tumour cell proliferation and regulation of epithelial to mesenchymal transition (EMT)-related phenotypes through mechanisms that are not fully understood. The essential mitochondrial import protein coiled-coil helix coiled-coil helix domain-containing protein 4 (CHCHD4) controls respiratory chain complex activity and oxygen consumption, and regulates the growth of tumours in vivo. In this study, we interrogate the importance of CHCHD4-regulated mitochondrial metabolism for tumour cell proliferation and EMT-related phenotypes, and elucidate key pathways involved.

**Results:**

Using in silico analyses of 967 tumour cell lines, and tumours from different cancer patient cohorts, we show that *CHCHD4* expression positively correlates with OXPHOS and proliferative pathways including the mTORC1 signalling pathway. We show that *CHCHD4* expression significantly correlates with the doubling time of a range of tumour cell lines, and that CHCHD4-mediated tumour cell growth and mTORC1 signalling is coupled to respiratory chain complex I (CI) activity. Using global metabolomics analysis, we show that CHCHD4 regulates amino acid metabolism, and that CHCHD4-mediated tumour cell growth is dependent on glutamine. We show that CHCHD4-mediated tumour cell growth is linked to CI-regulated mTORC1 signalling and amino acid metabolism. Finally, we show that *CHCHD4* expression in tumours is inversely correlated with EMT-related gene expression, and that increased CHCHD4 expression in tumour cells modulates EMT-related phenotypes.

**Conclusions:**

CHCHD4 drives tumour cell growth and activates mTORC1 signalling through its control of respiratory chain mediated metabolism and complex I biology, and also regulates EMT-related phenotypes of tumour cells.

**Electronic supplementary material:**

The online version of this article (10.1186/s40170-019-0200-4) contains supplementary material, which is available to authorized users.

## Introduction

Rapidly dividing tumour cells require specific metabolites to support proliferation, and the metabolic rewiring of malignant cells contributes both to transformation and tumour progression [1]. One of the earliest observations of metabolic adaptation in tumour cells came from the work of Otto Warburg, who identified that even in the presence of sufficient oxygen, many cancer cells consumed high concentrations of glucose and secreted high levels of lactate [2]. In addition to oncogene-driven increases in glucose consumption, tumour cells also increase their consumption of glutamine [3], as glutamine provides carbon and nitrogen moieties for amino acid and nucleotide synthesis. Despite the prevalence of oxidative fermentation in transformed cells, tumour cells retain their oxidative mitochondrial machinery to support the catabolism of glucose and glutamine for the production of macromolecules to permit cell division [4, 5]. Indeed, a variety of recent studies have demonstrated that while the availability of adenosine triphosphate (ATP) is unlikely to be a limiting factor for the proliferation of tumour cells [6–8], the availability of amino acids and nucleotides can be important depending on the cellular context [8–12]. However, our understanding of the mechanisms by which transformed cells maintain biosynthesis of macromolecules for cell division remains incomplete. Delineating the pathways which support tumour cell proliferation are of great interest for the development of new therapeutic strategies, and anti-tumour agents which inhibit nucleotide synthesis (e.g. fluorouracil) have been used clinically for decades [13]. Therapies which target amino acid synthesis are emerging in clinical development, and are showing promise [14–16].

Mitochondria support cellular proliferation by supplying ATP for the bioenergetic demands of the cell through OXPHOS, and are also the site of reactions which supply the cell with precursors for the synthesis of macromolecules such as DNA, proteins and lipids [17]. In addition, complex I (CI) of the respiratory chain regulates the intracellular NADH/NAD ratio, which is itself an essential cofactor for biosynthetic reactions which support proliferation [18]. Depletion of mitochondrial DNA in tumour cells (ρ^0^ cells) inhibits tumour cell proliferation and tumorigenesis in vitro and in vivo, demonstrating the importance of mitochondria in a cancer setting [19–21]. Furthermore, CI-inhibiting biguanidines (e.g. metformin) are effective in slowing the growth of tumour cells in vitro [22, 23], and are under investigation in clinical trials as adjuvant therapies for cancer patients.

All but 13 subunits of the respiratory chain complexes are encoded by nuclear genes, and must be imported across the mitochondrial membrane(s) for complex assembly to take place. Several import and sorting pathways exist in the mitochondria which are essential for respiratory chain activity [24]. One such pathway, the disulfide relay system (DRS), is responsible for the import and oxidative folding of small intermembrane space (IMS) proteins, which include subunits of CI and CIV, as well as assembly factors for CIII and CIV [25–27]. The substrate-binding oxidoreductase of the DRS is the redox-sensitive protein CHCHD4, which we have shown to regulate cellular oxygen consumption rate, and hypoxia-inducible factor (HIF) signalling [28–30]. We have shown that increased *CHCHD4* expression in tumours correlates with increased tumour progression, and is associated with decreased patient survival and disease recurrence [28]. In this study, we investigate the molecular mechanisms by which CHCHD4 controls tumour cell proliferation through its effects on mitochondrial metabolism. Using gene set enrichment analysis (GSEA) to identify key tumour-related pathways associated with *CHCHD4* expression that are linked to OXPHOS and tumour proliferation, we investigate the role of CHCHD4 in regulating tumour cell growth, mTORC1 signalling, amino acid metabolism and EMT-related phenotypes.

## Results

### *CHCHD4* expression positively correlates with OXPHOS and proliferative pathways in tumours

**Fig. 1:**
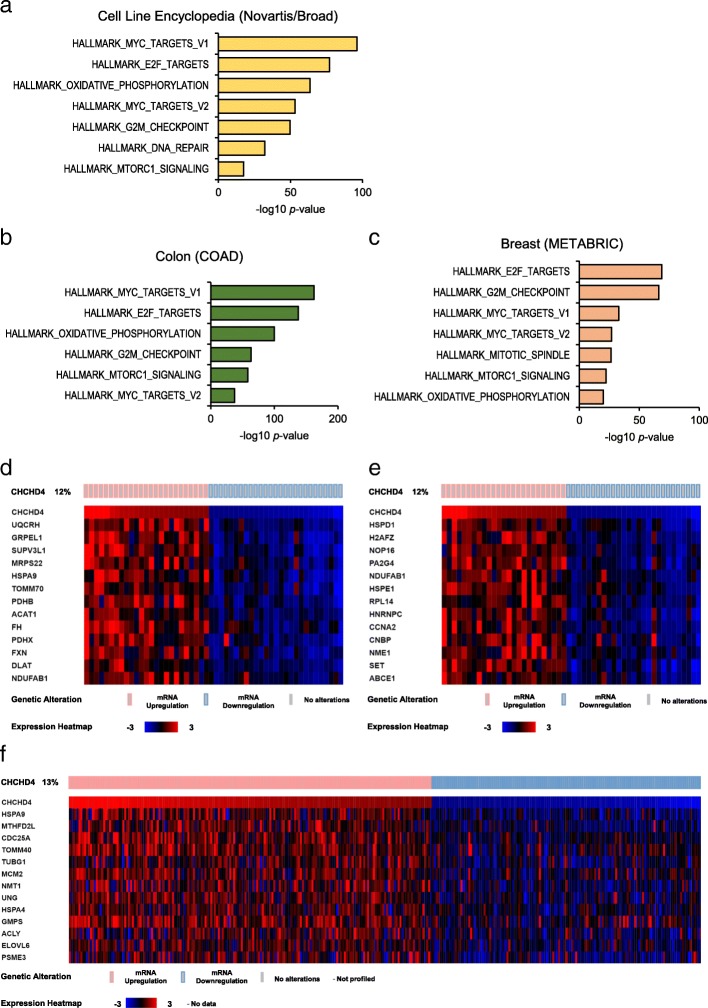
*CHCHD4* expression positively correlates with OXPHOS and proliferative pathways in tumours. **a** Chart shows GSEA of genes positively correlated with *CHCHD4* expression in Novartis/Broad Institute Cancer Cell Line Encyclopedia RNASeq data. *n* = 967 cell lines. **b**, **c** Charts show GSEA of genes positively correlated with *CHCHD4* expression in colon adenocarcinoma (**b**) and breast cancer (**c**) patient tumours. **d** Heatmap of selected genes from the HALLMARK_OXIDATIVE_PHOSPHORYLATION gene-set (Broad Institute) that are positively correlated with *CHCHD4* expression in colon adenocarcinoma patient tumours. **e**, **f** Heatmap of selected genes from the HALLMARK_MTORC1_SIGNALLING gene-set (Broad Institute) that are positively correlated with *CHCHD4* expression in colon adenocarcinoma (**e**) and breast cancer (**f**) patient tumours

Recently, we have shown that elevated CHCHD4 expression in tumour cells increases tumour cell proliferation irrespective of oxygen levels [30]. To better understand the relationship between CHCHD4 and tumour cell proliferation, we carried out GSEA on genes that were significantly correlated with *CHCHD4* expression in transcriptomic data from a panel of 967 tumour cell lines (Novartis/Broad Institute Tumour Cell Line Encyclopaedia). As anticipated, *CHCHD4* was significantly co-expressed with genes from an OXPHOS gene set (HALLMARK_OXIDATIVE_PHOSPHORYLATION, Broad Institute) (Fig. 1a), including subunits of CI (*NDUFS3*) and CIV (*COX7C*) (Additional file 1: Figure S1a). Interestingly, many of the most significantly enriched gene sets correlated with *CHCHD4* expression were those from proliferative signalling pathways regulated by known oncogenes, such as MYC and mTORC1 (Fig. 1a). Genes regulated by mTORC1 signalling that were most significantly correlated with *CHCHD4* expression include cell-cycle regulators (*CDC25A, CCNF*) and subunits of DNA polymerases (*POLR3G*) (Additional file 1: Figure S1b). We next carried out GSEA on genes that were significantly co-expressed with *CHCHD4* in tumours from different cancer patient cohorts using transcriptomic data available from The Cancer Genome Atlas (http://portal.gdc.cancer.gov/). Again, we found that *CHCHD4* expression was significantly associated with genes involved in OXPHOS in colon adenocarcinoma (Fig. 1b), breast cancer (Fig. 1c) and glioblastoma (Additional file 1: Figure S1c) patient samples. In colon adenocarcinoma (Fig. 1d) and breast cancer patient samples (Fig. 1e), the most highly correlated genes involved in OXPHOS constituted components of respiratory chain complexes (e.g. *UQCRH*, *NDUFAB1*), as well as enzymes of the TCA cycle (*DLAT*, *FH*), and a variety of other genes that encode proteins involved in general maintenance of mitochondrial function (e.g. *TOMM70*, *HSPA9*). As we observed for our GSEA of tumour cell lines (Fig. 1a), *CHCHD4* expression was also most highly correlated with the expression of genes regulated by proliferative signalling pathways including MYC and mTORC1, in tumours from each of the cancer patient cohorts analysed (Fig. 1b, c, Additional file 1: Figure S1c). Genes regulated by mTORC1 signalling that were associated with *CHCHD4* expression in colon adenocarcinoma patient samples included cell cycle regulators (*CCNA2, CDC25A*), and genes that encode proteins involved in nucleotide synthesis (*MTHFD2L*). Together, these data indicate that across a broad range of tumour cell lines and diverse tumour types, *CHCHD4* expression is positively associated with OXPHOS, and key proliferative pathways such as the mTORC1 signalling pathway.

### CHCHD4-mediated tumour cell growth and mTORC1 signalling is coupled to CI activity

**Fig. 2:**
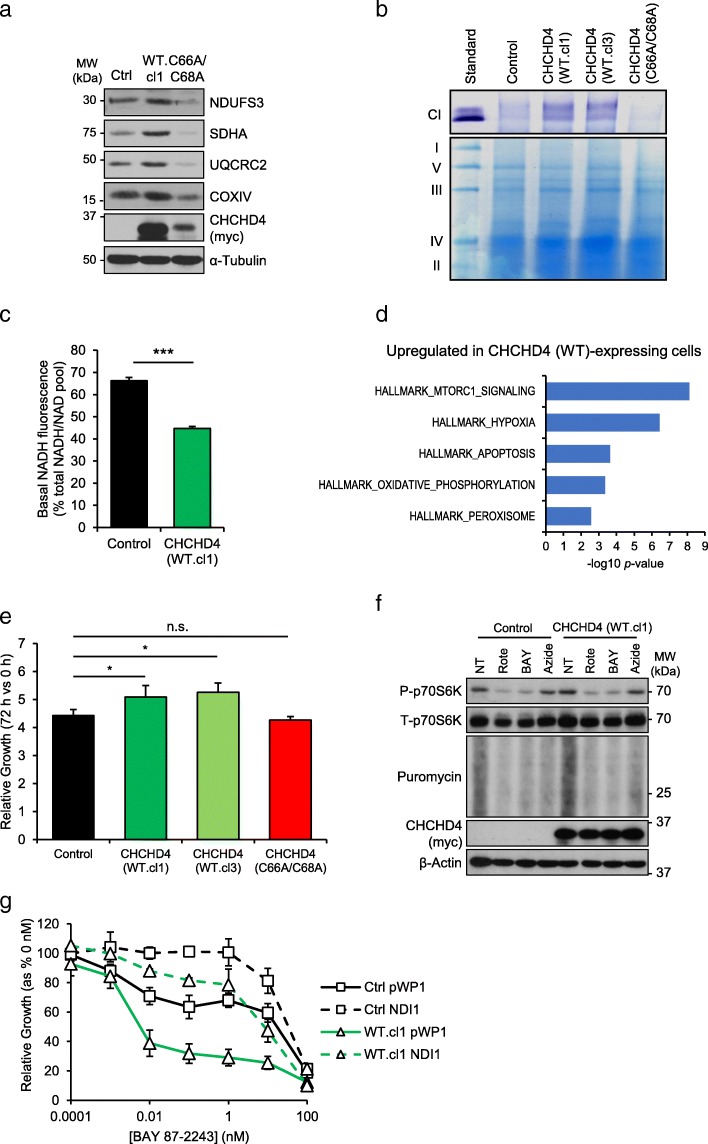
CHCHD4-mediated tumour cell growth and mTORC1 signalling is coupled to CI activity. **a** Western blots show levels of CHCHD4, NDUFS3 (CI), SDHA (CII) UQCRC2 (CIII), COXIV (CIV) in whole cell lysates of control (Ctrl) U2OS cells, CHCHD4 (WT)-expressing cells (WT.cl1) or CHCHD4-(C66A/C68A)-expressing cells (C66A/C68A). α-Tubulin was used as a load control. **b** In-gel NTB assay of CI activity in mitochondrial fractions isolated from control U2OS cells, CHCHD4 (WT)-expressing cells (WT.cl1, WT.cl3) and CHCHD4 (C66A/C68A)-expressing cells (C66A/C68A). **c** Basal NADH fluorescence (as % of total NADH/NAD pool) in control U2OS cells or CHCHD4 (WT)-expressing cells (WT.cl1). ± SD. *n* = 3. **d** Chart shows GSEA of proteins upregulated in CHCHD4 (WT)-expressing cells (WT.cl1) relative to control U2OS cells, as assessed by SILAC [30]. *n* = 3. **e** Chart shows relative growth rates of cells described in (**b**) at 72 h relative to 0 h. ± SD. *n* = 3. **f** Western blots show levels of phosphorylated (P-) and total (T-) p70S6K, and puromycin labelled polypeptides in control U2OS cells and CHCHD4 (WT)-expressing cells (WT.cl1) either untreated (NT), or treated with rotenone (500 nM), BAY 87-2243 (50 nM) or sodium azide (100 μM) for 24 h. β-Actin was used as a load control. **g** Chart shows relative growth of control U2OS cells, and CHCHD4 (WT)-expressing cells (WT.cl1) stably expressing an empty vector (pWPI) or yeast NDI1, treated with BAY 87-2243 using a 10-fold dilution curve (starting dose 100 nM) for 72 h. ± SD. *n* = 3

Our in silico analyses demonstrated a positive association between *CHCHD4* expression, OXPHOS and proliferative pathways in tumour cell lines and patient tumour samples (Fig. 1). Thus, we next investigated the relationship between CHCHD4-mediated regulation of OXPHOS and tumour cell growth. To do this, we used U2OS cells stably expressing wild-type CHCHD4 [CHCHD4 (WT)-expressing cells, clones WT.cl1, WT.cl3], or stably expressing a mutant form of CHCHD4 in which the substrate-binding cysteines of the CPC motif have been mutated to alanines (C66A/C68A) described by us previously [28–30]. Consistent with our previous study [30], we found that elevated CHCHD4 expression led to increased expression of individual subunits of CI (NDUFS3), CII (SDHA), CIII (UQCRC2) and CIV (COXIV) (Fig. 2a). Importantly, expression of the C66A/C68A mutant form of CHCHD4, which we and others have shown is defective in import function and mitochondrial localisation [28, 31], did not lead to increased mitochondrial expression of these respiratory chain subunits (Fig. 2a). Notably, we and others have shown that loss of CHCHD4 leads to reduced levels of apoptosis-inducing factor (AIF) [30, 32, 33], a CHCHD4-interacting protein [33]. However, we found no obvious effect on the expression of AIF in CHCHD4 overexpressing cells (Additional file 2: Figure S2a).

We and others have shown that CHCHD4 is a critical regulator of CI expression [30, 33]. Further examination of CI activity using an in-gel nitrotetrazolium blue assay demonstrated increased CI activity in mitochondrial extracts from CHCHD4 (WT)-expressing cells (WT.cl1, WT.cl3), while whole CI activity was reduced in CHCHD4 (C66A/C68A)-expressing cells, compared to control U2OS cells (Fig. 2b). CI catalyses the first step of NADH oxidation, providing the cell with NAD^+^ which acts as an essential cofactor for TCA cycle enzymes, and enzymes involved in amino acid and nucleotide biosynthesis. Using live cell imaging of NADH fluorescence [34] (Additional file 2: Figure S2b), we found that CHCHD4 (WT)-expressing cells exhibited lower basal NADH fluorescence compared with control U2OS cells (Fig. 2c, Additional file 2: Figure S2b), indicating higher CI activity in live cells with elevated CHCHD4 expression, consistent with our in-gel assay for CI activity (Fig. 2b).

Using stable isotope labelling with amino acid in cell culture (SILAC) and proteomic analysis of CHCHD4 (WT)-expressing cells, we have recently shown that CHCHD4 expression in tumour cells is a critical determinant of the expression of a broad range of respiratory chain subunits including numerous subunits of CI [30]. Analysis of our SILAC data showed the most significantly enriched proteins in CHCHD4 (WT)-expressing cells compared to control cells were those regulated by the mTORC1 signalling pathway (e.g. GPI, E2F1) (Fig. 2d), consistent with our GSEA of tumour cell lines and patient tumour samples (Fig. 1a–c). As mTORC1 is a potent regulator of proliferation [35], we next assessed the effects of exogenous expression of CHCHD4 on the proliferation rates of U2OS cells. Consistent with our recently published study [30], we found that elevated expression of CHCHD4 in the CHCHD4 (WT)-expressing cells led to a small but significant increase in growth rates over 72 h compare to control U2OS cells (Fig. 2e). However, exogenous expression of mutant (C66A/C68A) CHCHD4 did not affect growth rate (Fig. 2e).

We hypothesised that CHCHD4 may regulate a CI-mTORC1-proliferation axis, and so we next investigated the influence of CHCHD4 expression on mTORC1 signalling in the absence or presence of respiratory chain inhibitors. The mTORC1 signalling pathway regulates cell growth and protein synthesis in part by directly phosphorylating p70S6 kinase 1 (p70S6K) [35]. Both basal p70S6K and protein translation rates (as assessed by puromycin incorporation) were consistently elevated in CHCHD4 (WT)-expressing cells compared to control cells (Fig. 2f) indicating higher basal mTORC1 activity. mTORC1 signalling was blocked by the GI_50_ dose of the CI inhibitors, rotenone (500 nM) and BAY 87-2243 (50 nM), while only puromycin incorporation was sensitive to inhibition of CIV (Fig. 2f, Additional file 2: Figure S2c), suggesting that CI and CIV influence protein translation through distinct mechanism(s). Consistently, we have shown that CHCHD4 expression confers increased sensitivity to growth inhibition by inhibitors of CI, such as rotenone and BAY 87-2243 (Fig. 2g) [30, 36], but not inhibitors of CIV [30]. To investigate whether the increased sensitivity conferred by CHCHD4 overexpression was due specifically to the loss of the NADH dehydrogenase activity of CI, we generated pools of control U2OS and CHCHD4 (WT)-expressing cells stably expressing a rotenone-insensitive NADH dehydrogenase from yeast (NDI1), or the empty expression vector (pWPI) (Additional file 2: Figure S2d). NDI1 expression was capable of significantly reducing the sensitivity of both control U2OS cells (*p* = 0.005) and CHCHD4 (WT)-expressing cells (*p* = 0.001) to growth inhibition in the presence of BAY 87-2243 (Fig. 2g). Importantly, NDI1 expression rendered CHCHD4 (WT)-expressing cells resistant to BAY 87-2243 to the same degree as control U2OS (pWPI expressing) cells (*p* = 0.09) (Fig. 2g). This indicates that the increased sensitivity of CHCHD4 expressing cells to CI inhibitors is dependent on the loss of NADH dehydrogenase activity in these cells. Collectively, our data suggest CHCHD4-mediated tumour cell growth and mTORC1 signalling is coupled to CI activity.

### CHCHD4 expression links growth rate to CI activity and mTORC1 signalling in tumour cells

**Fig. 3:**
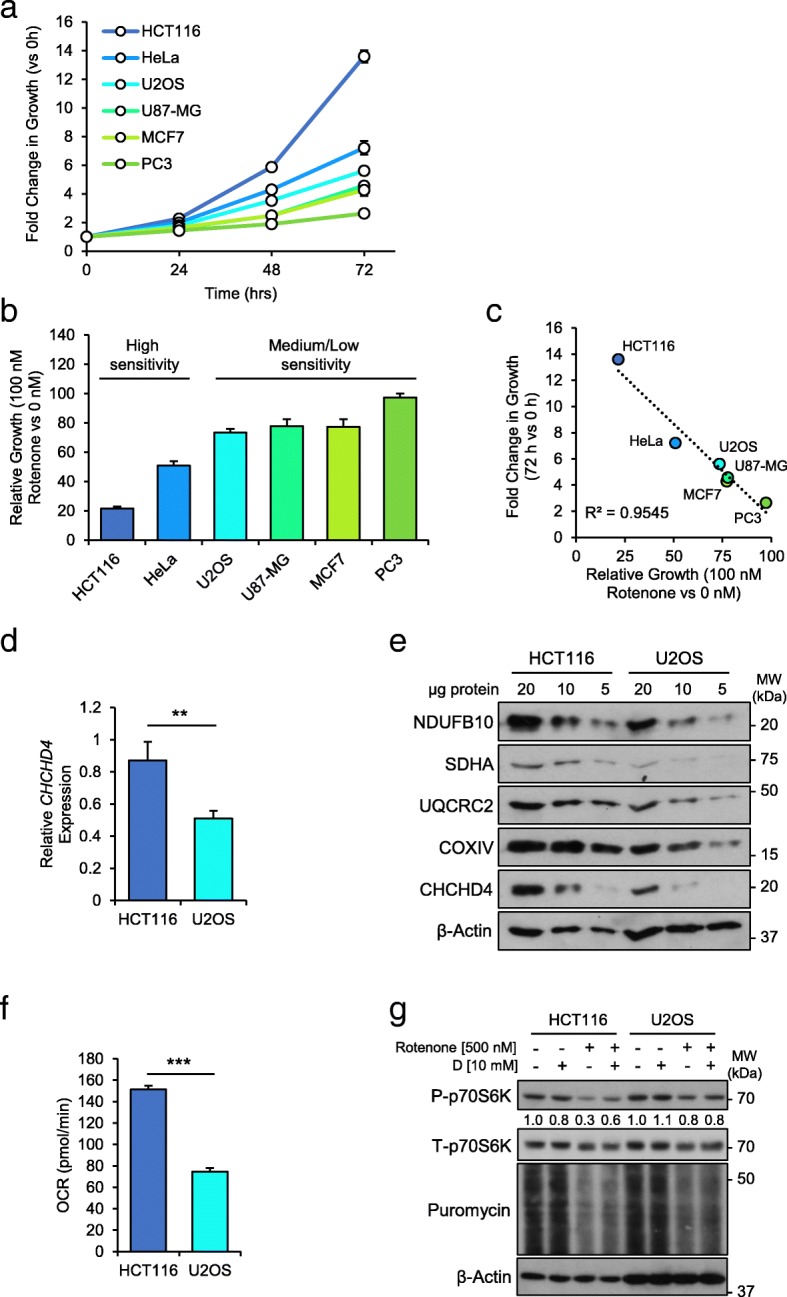
CHCHD4 expression links growth rate to CI activity and mTORC1 signalling in tumour cells. **a** Chart shows the growth rates of a panel of tumour cell lines over 72 h. ± SD. *n* = 3. **b** Chart shows growth of tumour cell lines described in (**a**) treated with 100 nM rotenone for 72 h, relative to untreated (0 nM) cells. ± SD. *n* = 3. **c** Chart shows xy scatter of the fold change in growth of indicated tumour cell lines at 72 h vs 0h, compared to their relative growth rate when treated with 100 nM rotenone. Trend line (dashed blue) and *R*^2^ value (Spearman’s correlation) shown. **d** Chart shows relative abundance of *CHCHD4* transcripts measured by QPCR in HCT116 and U2OS cells. *CHCHD4* expression is relative to *ACTB* and normalised to CHCHD4 transcript levels in HCT116 cells. ± SD. *n* = 3. **e** Western blots show levels of CHCHD4, NDUFB10 (CI), SDHA (CII) UQCRC2 (CIII), COXIV (CIV) in whole cell lysates of HCT116 and U2OS cells. β-Actin was used as a load control. **f** Chart shows basal OCR of HCT116 and U2OS cells, measured by Seahorse respirometry. ± SD. *n* = 3. **g** Western blots show levels of phosphorylated (P-) and total (T-) p70S6K, puromycin labelled polypeptides in HCT116 and U2OS cells treated as indicated for 24 h. β-Actin was used as a load control. Relative band intensities of P-p70S6K indicated

To further investigate the positive association between CHCHD4 expression, CI and mTORC1 signalling (Figs. 1 and 2), we next examined a panel of tumour cell lines to determine whether cells exhibited differing dependencies on CI activity for growth. To do this, we selected six well-known tumour cell lines derived from different tissues (colon, cervix, bone, breast, brain and prostate), each with a different genetic background, and each exhibiting a different relative growth rate (Fig. 3a). To characterise the dependency of these cell lines on OXPHOS for growth, we assessed their relative sensitivity to respiratory chain inhibition using titrations of the CI inhibitors, rotenone (Fig. 3b) and BAY 87-2243 (Additional file 3: Figure S3a), and the CIII inhibitor antimycin A (Additional file 3: Figure S3b). Sensitivity to these agents was inversely correlated with the relative growth rates of these cell lines (Fig. 3c), demonstrating the common importance of respiratory chain activity for tumour cell growth. We next assessed CHCHD4 expression levels in two cell lines from our panel, one with a high growth rate and increased sensitivity to rotenone (HCT116 colon carcinoma, GR_50_ = 42.4 ± 6.3 nM), and one with a lower growth rate and reduced sensitivity to respiratory chain inhibition (U2OS osteosarcoma, GR_50_ = 341 ± 101 nM). Interestingly, CHCHD4 expression levels were higher in HCT116 cells compared to U2OS cells both at the transcript level (Fig. 3d) and protein level (Fig. 3e). Furthermore, expression of subunits from each of the respiratory chain complexes (CI-IV) was higher in HCT116 cells compared to U2OS cells (Fig. 3e), as was basal oxygen consumption rate (Fig. 3f), demonstrating increased respiratory chain activity. These data indicate a link between CHCHD4 expression levels, OXPHOS and tumour cell growth across a range of tumour cell types with different aetiologies and oncogenic drivers. In fact, we also found that *CHCHD4* expression was weakly but significantly inversely correlated with the doubling times of 368 different tumour cell lines from the Cancer Cell Line Encyclopedia (CCLE, Broad Institute) (Additional file 3: Figure S3c).

As our GSEA of tumour cell lines identified the mTORC1 signalling pathway as positively associated with *CHCHD4* expression (Fig. 1a), next we compared the effects of respiratory chain inhibition on mTORC1 signalling in HCT116 and U2OS cells. Basal mTORC1 signalling was not apparently different between HCT116 and U2OS cells as assessed by p70S6K phosphorylation, and puromycin incorporation (Fig. 3g). Consistently there was a larger reduction in both p70S6K phosphorylation and puromycin incorporation in HCT116 cells with rotenone treatment compared to U2OS cells (Fig. 3g). These data indicate that mTORC1 signalling in HCT116 cells is more sensitive to CI inhibition (Fig. 3g), similar to U2OS cells overexpressing CHCHD4 (Fig. 2f), further supporting a link between CHCHD4 expression, CI and mTORC1 signalling. It is known that CI, through the production of NAD^+^ from the oxidation of NADH, can promote the synthesis of asparagine which directly activates mTORC1 signalling [35], and that aspartate, which is required for the de novo synthesis of nucleotides, can also directly activate mTORC1 signalling [37, 38]. Indeed, we found that supplementation of aspartate (D) to the media of both HCT116 and U2OS cells partially rescued the reduced mTORC1 signalling observed in the presence of rotenone (Fig. 3g). Collectively, our data suggest that CHCHD4 expression links growth rate to CI activity, mTORC1 signalling and amino acid metabolism in tumour cells.

### CHCHD4-mediated tumour cell growth is linked to CI-regulated mTORC1 signalling and amino acid metabolism

**Fig. 4:**
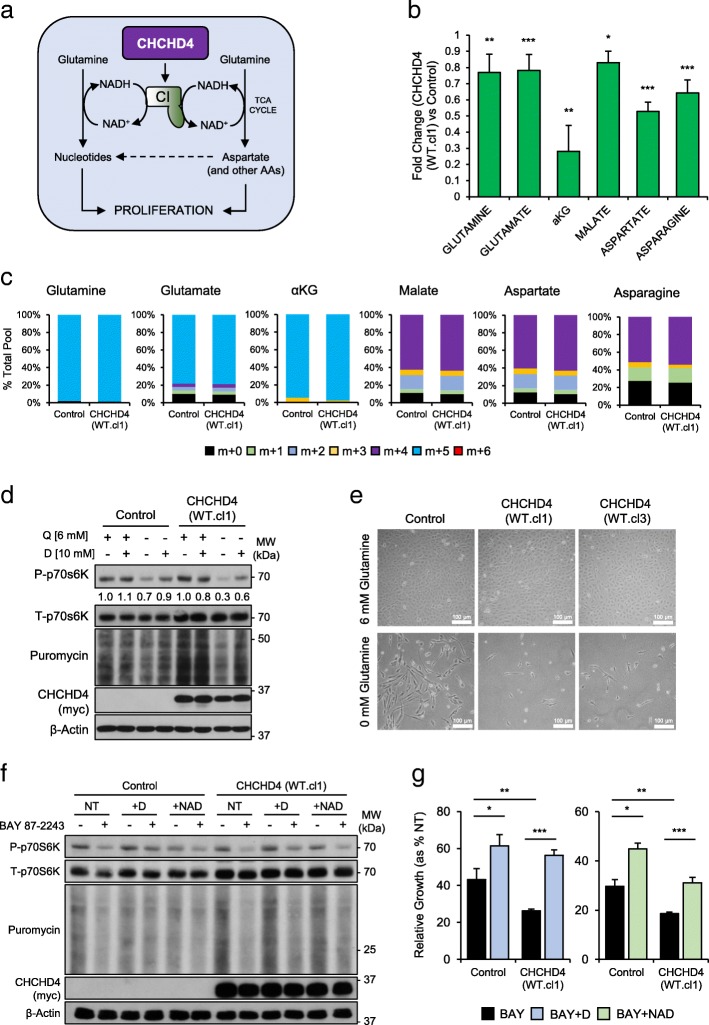
CHCHD4-mediated tumour cell growth is linked to CI-regulated mTORC1 signalling and amino acid metabolism. **a** Schematic of proposed model of CHCHD4-regulated CI-dependent amino acid (AA) metabolism and influence on tumour cell growth. **b** Chart shows relative intracellular abundance of selected metabolites from metabolomics analysis in CHCHD4 (WT)-expressing cells (WT.cl1) relative to control U2OS cells. Representative of 2 experiments. ± SD. *n* = 5. **c** Chart shows relative proportions of isotopically labelled metabolites in control U2OS cells and CHCHD4 (WT)-expressing cells (WT.cl1). Representative of 2 experiments. *n* = 5. **d** Western blots show levels of phosphorylated (P-), total (T-) p70S6K and puromycin labelled polypeptides in control U2OS cells and CHCHD4 (WT)- expressing cells (WT.cl1) treated as indicated for 8 h. β-Actin was used as a load control. Relative band intensities of P-p70S6K indicated. **e** Images of control U2OS cells and CHCHD4 (WT)-expressing cells (WT.cl1, WT.cl3) cultured to confluency in the presence of glutamine (6 mM), and 3 days later following removal of glutamine (0 mM). **f** Western blots show levels of phosphorylated (P-) and total (T-) p70S6K, and puromycin labelled polypeptides in control U2OS cells and CHCHD4 (WT)-expressing cells (WT.cl1) treated either in the absence (−) or presence (+) of 50 nM BAY 87-2243 for 24 h, supplemented with 10 mM aspartate (D) or 1 mM NAD. β-Actin was used as a load control. **g** Charts show relative growth of control U2OS cells and CHCHD4 (WT)-expressing cells (WT.cl1) treated with 10 nM BAY 87-2243 in the absence or presence of 10 mM aspartate (D) or 1 mM NAD for 72 h. ± SD. *n* = 3

Based on our data thus far (Figs. 1, 2 and 3), we hypothesised that CHCHD4-mediated regulation of respiratory chain activity stimulates tumour cell growth through the promotion of biosynthetic pathways. In the case of CI, increased activity promotes favourable NAD/NADH ratios for the biosynthesis of amino acids such as aspartate via the TCA cycle (Fig. 4a). Our NADH measurements showed decreased basal intracellular NADH levels (relative to the total NADH/NAD pool) (Fig. 2c, Additional file 2: Figure S2b) alongside increased CI activity in CHCHD4 (WT)-expressing cells compared to control U2OS cells (Fig. 2b). We therefore next carried out metabolomics analysis to assess the effects of CHCHD4 expression on the cellular metabolome, with particular focus on TCA cycle intermediates and amino acids. CHCHD4 (WT)-expressing cells and control U2OS cells were cultured with uniformly labelled ^13^C5-glutamine, followed by analysis of both the isotopologue composition and total pool sizes of key glutamine-derived metabolites. We found that intracellular levels of glutamine and other glutamine-derived metabolites were lower in CHCHD4 (WT)-expressing cells compared to control U2OS cells (Fig. 4b). Conversely, the extracellular levels of glutamine were not significantly different (Additional file 4: Figure S4), suggesting that rates of intracellular glutamine consumption were higher in CHCHD4 (WT)-expressing cells compared to control U2OS cells. There were only very minor differences in the isotopologue composition of a selection of these metabolites (Fig. 4c), indicating that there was no significant change in the routes of glutamine (and other amino acid) consumption in CHCHD4 (WT)-expressing cells compared to control U2OS cells. We hypothesised that by increasing CI expression and activity, elevated CHCHD4 expression was stimulating amino acid biosynthesis, leading to increased mTORC1 activity, protein synthesis rates and thus increased consumption of glutamine-derived amino acids (such as aspartate and asparagine). We therefore next tested whether glutamine was required for the increased mTORC1 signalling and protein synthesis in CHCHD4 (WT)-expressing cells. Glutamine withdrawal led to a larger reduction in both p70S6K phosphorylation and puromycin incorporation in CHCHD4 (WT)-expressing cells (Fig. 4d), indicating that glutamine metabolism was required to support the increased mTORC1 activity in these cells. Indeed, supplementation with aspartate was capable of partially rescuing mTORC1 activity on withdrawal of glutamine (Fig. 4d). Furthermore, CHCHD4 (WT)-expressing cells were lost more rapidly from culture on withdrawal of glutamine than control U2OS cells, indicating a higher dependency on glutamine for their proliferation and survival (Fig. 4e). As we hypothesised that the CI-mediated stimulation of amino acid metabolism may in part underpin the increased mTORC1 activity and proliferation of CHCHD4 (WT)-expressing cells (Fig. 4a), we next assessed whether aspartate or NAD supplementation could rescue mTORC1 activity and the proliferation of cells treated cells with the CI inhibitor BAY 87-2243. Both aspartate and NAD supplementation were capable of partially rescuing p70S6K phosphorylation and puromycin incorporation in the presence of BAY 87-2243 (Fig. 4f). Furthermore, both aspartate and NAD (Fig. 4g) were capable of partially rescuing the growth of both CHCHD4 (WT)-expressing cells and control U2OS cells treated with BAY 87-2243. Interestingly, while CHCHD4 (WT)-expressing cells were more sensitive to BAY 87-2243 treatment, both aspartate and NAD supplementation were able to more significantly rescue the growth of CHCHD4 (WT)-expressing cells compared to control U2OS cells treated with BAY 87-2243 (Fig. 4g). Together, these data demonstrate that CHCHD4 expression regulates amino acid metabolism, which in part underpins CHCHD4-mediated tumour cell growth and CI-regulated mTORC1 signalling.

### CHCHD4 regulates the EMT phenotype of tumour cells

**Fig. 5:**
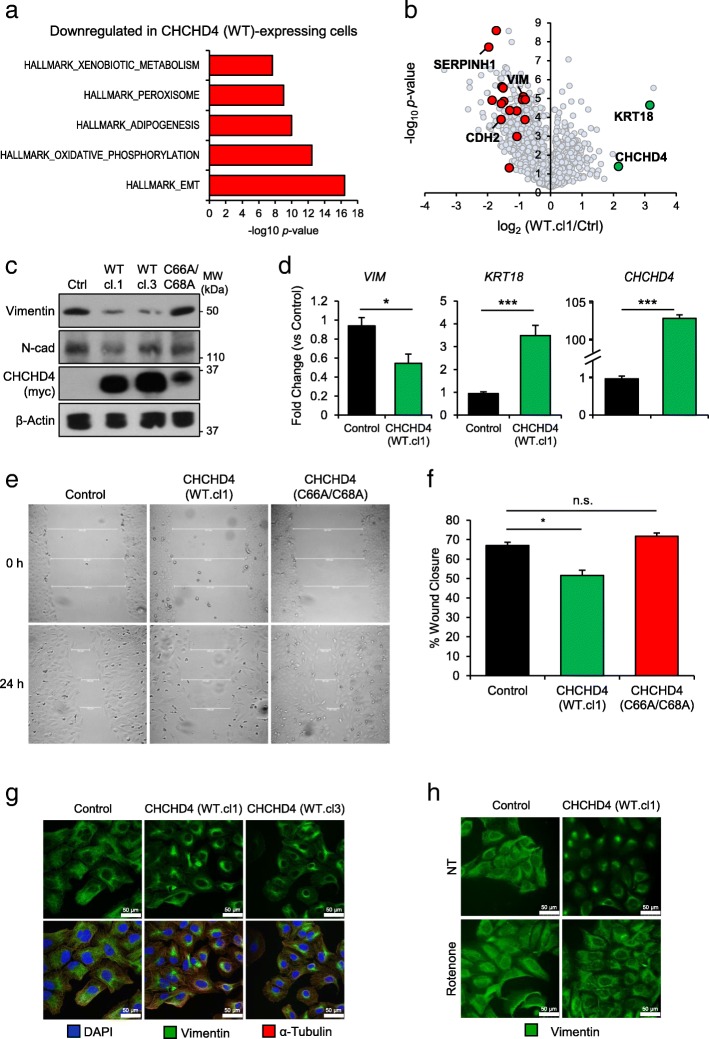
CHCHD4 regulates the EMT phenotype of tumour cells. **a** Chart shows GSEA of downregulated proteins detected in SILAC analysis of CHCHD4 (WT)-expressing cells (WT.cl1), compared to control U2OS cells. *n* = 3. **b** Volcano plot shows relative expression of all proteins detected in SILAC analysis of CHCHD4 (WT)-expressing cells (WT.cl1) compared to control (Ctrl) U2OS cells [30]. Selected genes highlighted. *n* = 3. **c** Western blots show levels of vimentin and N-cadherin in control (Ctrl) U2OS cells, CHCHD4 (WT)-expressing cells (WT.cl1, WT.cl3) and CHCHD4 (C66A/C68A)-expressing cells (C66A/C68A). β-Actin was used as a load control. **d** Chart shows relative transcript levels of *VIM*, *KRT18* and *CHCHD4* detected by Q-PCR in control U2OS cells and CHCHD4 (WT)-expressing cells (WT.cl1). ± SD. *n* = 3. **e** Images show a scratch-wound assay of control (Ctrl) U2OS cells, CHCHD4 (WT)-expressing cells (WT.cl1) and CHCHD4 (C66A/C68A)-expressing cells (C66A/C68A) at 0 h and 24 h. White lines denote width of scratch-wound. **f** Chart shows % wound closure at 24 h relative to 0 h in images shown in (**e**). ± SD. *n* = 3. **g** Immunofluorescence images of vimentin distribution in control U2OS cells and CHCHD4 (WT)-expressing cells (WT.cl1, WT.cl3). **h** Immunofluorescence images of vimentin distribution in control U2OS cells and CHCHD4 (WT)-expressing cells (WT.cl1) either untreated (NT) or treated with 50 nM rotenone for 72 h

Along with identifying significantly upregulated gene profiles associated with increased *CHCHD4* expression (Fig. 1), interestingly, our GSEA also showed that (EMT)-related genes were amongst the most significantly downregulated genes associated with increased *CHCHD4* expression. This was also the case in our SILAC dataset from CHCHD4 (WT)-expressing cells (Fig. 5a, b), as well as our analysis of transcriptomic data from patient samples (Additional file 5: Figure S5a, b) and tumour cell lines (Additional file 5: Figure S5c). Downregulated EMT-related genes included those encoding the essential cytoskeletal protein vimentin, and the extracellular matrix (ECM) binding protein N-cadherin (CDH2), both of which are well-characterised markers of EMT (Fig. 5b) [39]. Furthermore, the intermediate cytoskeleton filament and mesenchymal-epithelial transition (MET) marker keratin-18 (KRT18, [40]) was the second most upregulated protein identified in our SILAC analysis (Fig. 5b). We confirmed some of these EMT protein expression changes in CHCHD4 (WT)-expressing cells, while these proteins were unchanged in CHCHD4 (C66A/C68A)-expressing cells compared to controls (Fig. 5c and Additional file 5: Figure S5d). We found similar results in HCT116 cells (which do not express detectable levels of vimentin [41]), where overexpression or silencing of CHCHD4 increased or decreased the expression of the MET marker E-cadherin respectively (Additional file 5: Figure S5e, f). Furthermore, we found that the transcript levels of *VIM* and *KRT18* were downregulated and upregulated respectively in cells overexpressing CHCHD4 (Fig. 5d), suggesting that the changes in EMT gene expression are at the level of transcription and that elevated CHCHD4 expression in tumour cells leads to a transcriptional suppression of EMT-related genes. Importantly, CHCHD4 (WT)-expressing cells had a lower migratory capacity in a 2D scratch-wound assay compared to both control and CHCHD4 (C66A/C68A)-expressing cells (Fig. 5e, f).

Recent studies have connected mitochondrial metabolism and health to the EMT phenotype of cultured cells, through changes in cytoskeletal organisation, adhesion and motility [42]. Indeed, it has long been appreciated that cell proliferation and motility are inversely related, both in physiology and disease [43–45]. Vimentin is an intermediate filament protein which regulates cell motility through association with other cytoskeletal proteins to promote pseudopodia formation at the cell periphery [46]. Immunofluorescent staining of vimentin and imaging of control U2OS, and CHCHD4 (WT)-expressing cells demonstrated that the cytoplasmic localisation of vimentin was affected by CHCHD4 expression, as it appeared to exhibit a more perinuclear distribution in CHCHD4 (WT)-expressing cells compared to control U2OS cells (Fig. 5g, Additional file 5: Figure S5 g). This redistribution of vimentin has previously been identified as a consequence of MET [47, 48]. To investigate whether the CHCHD4-mediated effects on changes in vimentin intracellular distribution were related to increased CI activity, we treated both control U2OS and CHCHD4 (WT)-expressing U2OS cells with rotenone, and assessed vimentin localisation by immunofluorescence. Rotenone treatment led to the redistribution of vimentin from the perinuclear region to the cytoplasm in CHCHD4 (WT)-expressing cells, phenocopying the vimentin distribution in control U2OS cells (Fig. 5h, Additional file 5: Figure S5g). Together, these data show that elevated CHCHD4 expression in tumour cells negatively correlates with EMT-related gene expression in normoxia, and reduces 2D cell migration. In addition, CHCHD4-mediated regulation of vimentin localisation is dependent on CI activity.

## Discussion

Dysregulated metabolism is a common feature of tumour cells, and numerous oncogenes are potent regulators of metabolic pathways that contribute to tumour cell proliferation. The mechanisms by which tumour cells maintain biosynthesis of macromolecules for division are incompletely understood and are currently the focus of intensive investigation. Here, we have identified CHCHD4 as a new regulator of the metabolic drive which supports tumour cell proliferation, in part through its effects on respiratory chain-mediated metabolism. We have also shown through in vitro and in silico analysis that CHCHD4 expression is positively associated with the activity of proliferative signalling pathways, such as the mTORC1 pathway in many different tumour cell lines and patient tumour samples. Additionally, our GSEA identified other proliferative pathways that were associated with *CHCHD4* expression, such as MYC and E2F. MYC is a well-characterised and commonly deregulated oncogene [49], is a potent regulator of mitochondrial metabolism and biogenesis [50], and like CHCHD4 collaborates with HIF signalling under certain contexts [51]. However, GSEA can be confounded by the promiscuity of common genes between different gene sets [52], and the mTORC1, MYC and E2F pathways are known to regulate the expression of common genes. Nevertheless, these analyses suggest that the influence of CHCHD4 on proliferation may impinge on multiple pathways with clinical relevance, which warrants further investigation.

Mitochondria promote proliferation in part through ATP synthesis, but tumour cells can adapt their metabolism to decrease their dependence on OXPHOS for ATP homeostasis, and upregulate non-mitochondrial pathways of ATP synthesis, such as glycolysis and anaerobic fermentation of pyruvate [53, 54]. Besides ATP synthesis, mitochondria are also important metabolic hubs for pathways that support biomass accumulation, and these pathways appear to be more acutely sensitive to perturbations in respiratory chain function than ATP synthesis. Indeed, here, we found that doses of the CI inhibitor BAY 87-2243 that are insufficient to block OCR [30] were capable of significantly influencing protein translation and tumour cell proliferation. In support of the essential role of mitochondrial metabolism for biomass accumulation, previous studies have demonstrated that the synthesis of aspartate is an essential function of mitochondrial respiration for proliferating cells [8, 10]. In agreement with these studies, we show here that aspartate supplementation is capable of partially reversing the anti-proliferative effects of both CI inhibition and glutamine withdrawal, and stimulates mTORC1-mediated activation of translation. Since this rescue by aspartate supplementation was only partial, we conclude that other biosynthetic pathways that depend on respiratory chain activity must also contribute to tumour cell proliferation. For example, both (CI-derived) NAD and glutamine are directly involved in nucleotide synthesis [18, 55, 56], and nucleotide supplementation in addition to aspartate supplementation may be necessary to fully recapitulate the proliferation of cells treated with CI inhibitors or glutamine withdrawal. Moreover, pyrimidine synthesis through the uridine salvage pathway relies directly on respiratory chain activity, through the action of the mitochondrial enzyme dihydroorotate dehydrogenase (DHODH) [57]. In cells depleted of mitochondrial DNA (ρ^0^ cells), supplementation with uridine is necessary to rescue the loss of proliferative capacity [58], demonstrating that supporting nucleotide synthesis is an essential role of the mitochondria for proliferating cells. Indeed, transcriptome analysis of patient samples has identified that upregulation of nucleotide biosynthetic genes is one of the most common metabolic alterations across cancer types [52, 59].

Cancer can be considered a disease of two major phenotypes: dysregulated proliferation, and metastatic dissemination of transformed cells to distant sites. Metastasis is usually (but not always) accompanied by the acquisition by tumour cells of traits which decrease cell-cell interactions, and increase motility and resistance to killing by commonly used therapeutics [60]. In recent years, it has become appreciated that mitochondria-mediated proliferation and EMT phenotypes are inversely related, though regulated by common pathways [42, 44, 45, 61, 62]. For example, analysis of transcriptome data from patient tumour samples has identified that gene sets involved in OXPHOS are commonly changed in tumour tissues compared to normal tissue [52]. However, the direction of change was found to be heterogeneous, with upregulation of OXPHOS in 35% of cancer types, and downregulated in 25% of cancer types [52]. Interestingly, in those cancers with downregulation of OXPHOS, EMT-related genes were the most significantly upregulated cohort, and these changes were correlated with worse outcomes for patients [52]. In agreement with this, we found that *CHCHD4* expression is also positively correlated with OXPHOS and proliferative signalling pathways (e.g. mTORC1, MYC, E2F), while it is negatively correlated with an EMT gene signature in tumour cells, and in patient tumour samples. While our study adds to the body of evidence supporting this (negative) correlation, little is known about the mechanistic relationship between these two phenotypes, and further work will be required to understand whether CHCHD4 influences proliferation and EMT phenotypes through similar or distinct mechanisms, e.g. via mitochondrial metabolism.

Our results suggest a paradox with respect to the importance of CHCHD4 in cancer. While we show here that increased *CHCHD4* expression correlates with decreased EMT gene expression, we have already demonstrated that above median *CHCHD4* expression is correlated with increased tumour grade and decreased survival [28]. Since metastatic disease is a common feature of disease progression [60], how then can elevated *CHCHD4* be associated with worse outcomes for patients in certain cancers? The answer may come from our previous work which identified CHCHD4 as a critical regulator of HIF-mediated transcriptional responses to hypoxia [28, 29]. The tumour microenvironment appears to be the primary driver of EMT, since no recurrent mutations in EMT-regulating genes have been identified from genomic sequencing of tumour cells, unlike the myriad mutations in oncogenes and tumour suppressors which regulate proliferation [60]. One significant environmental stimulus of EMT is hypoxia, which is a common feature of tumour tissues which outgrow their vascular supply [63, 64]. Activation of HIF-signalling under hypoxia leads to transcriptional activation of EMT-related genes such as vimentin and N-cadherin, along with the suppression of MET-related genes such as E-cadherin [65]. Furthermore, HIF-signalling decreases mitochondrial OXPHOS by diverting carbon fuels away from metabolism by the mitochondria [53, 66], and in some cases decreases mitochondrial mass by suppressing mitochondrial biogenesis [67]. Thus, in tumours with elevated CHCHD4 expression, the increased proliferation it affords may increase the size of the hypoxic niche, while simultaneously enhancing HIF activation, and consequently metastatic dissemination of tumour cells. Indeed, we have shown that silencing of *CHCHD4* significantly decreases the hapto-migration and invasion of HCT116 cells in hypoxia [28], demonstrating the relationship between CHCHD4 and EMT phenotypes in hypoxia. It will be important to make more detailed investigations into the influence of CHCHD4 on hypoxia responses and metastatic phenotypes in 3D culture models and patient tumours, to fully understand the mechanisms by which CHCHD4 influence metastasis.

From a therapeutic perspective, mitochondrial metabolism is an attractive target for treatment regimes, but is not without significant risk of toxicity. Agents which target the mitochondria are potently anti-proliferative, and several small molecules are in clinical trials which inhibit mitochondrial metabolic pathways [14, 15, 23]. The present study points to a potential complication with this kind of therapeutic strategy however; in that targeting mitochondrial metabolism (e.g. at CI) may stimulate tumour cells to develop EMT characteristics which promote metastatic dissemination. Primary tumour cells which evade killing by chemotherapies may therefore contribute to the metastatic population of transformed cells, and anti-proliferative agents may in fact contribute to disease progression and/or relapse. It will be important to thoroughly understand the molecular mechanisms which underpin this apparent inverse relationship between proliferation and EMT in order to be able to devise treatment regimens which effectively and systemically remove transformed cells from patients.

## Conclusions

Mitochondrial metabolism plays a central role in tumour cell proliferation. Our current study demonstrates that the mitochondrial import protein CHCHD4 regulates tumour cell proliferation through its effects on CI expression and activity. Increased CI activity increases metabolism of glutamine, and drives mTORC1-mediated signalling. Furthermore, our study demonstrates that CHCHD4 expression regulates the EMT phenotype of tumour cells, and is negatively correlated with the expression of EMT genes in normoxia. Future studies will further investigate the influence of CHCHD4 on the metabolic landscape of cultured tumour cell lines (e.g. nucleotide synthesis), and the proliferation and metastatic behaviour of tumour cells in vitro and in vivo.

## Methods

### Cell culture

Human U2OS, HeLa, MCF7, HCT116, U87-MG and PC3 cell lines were all obtained from American Tissue Culture Collection (ATCC). Human osteosarcoma U2OS control and independent clonal cell lines (WT.cl1 and WT.cl3) expressing CHCHD4.1 cDNA (CHCHD4 (WT)-expressing cells) or CHCHD4-C66A/C68A cDNA (CHCHD4 (C66A/C68A)-expressing cells) have been described by us previously [29]. Human U2OS cells and CHCHD4 (WT.cl1)-expressing cells were transfected with either pWPI (IRES-EGFP) empty vector control or pWPI:Ndi1(IRES-EGFP) which have been described previously [68]. Both pWPI vectors were co-transfected with an empty puromycin-resistance cassette containing vector (pCMV6-A-Puro) for initial mammalian cell selection. Following puromycin selection and expansion of cell pools, pWPI-expressing cells were selected by flow cytometric sorting of GFP-positive cells. *Ndi1* (*NDI1*) expression was confirmed by PCR amplification, followed by agarose gel electrophoresis (Fig. S2d). All cell lines were maintained in Dulbecco’s modified eagle medium (DMEM) containing glucose (4.5 g/L) (Life Technologies), and supplemented with 10% fetal calf serum (FCS, SeraLabs), penicillin (100 IU/mL), streptomycin (100 μg/mL) and glutamine (6 mM), all purchased from Life Technologies. Cell lines used were authenticated and routinely confirmed to be negative for any mycoplasma contamination.

### Antibodies and reagents

The rabbit polyclonal P-p70S6K (#9205), T-p70S6K (##2708), LC3B (#3868), SDHA (#11998), COXIV (#4850), MYC-tag (2272), Vimentin (#3932) and N-cadherin (#13116) antibodies were purchased from Cell Signaling Technology. The rabbit polyclonal CHCHD4 (HPA034688) antibody was purchased from Cambridge Biosciences. The rabbit polyclonal NDUFB10 (ab196019), NDUFS3 (ab110246), UQCRC2 (ab14745) and mouse monoclonal α-Tubulin (ab7291) and β-actin (ab6276) antibodies were purchased from Abcam. The goat anti-mouse IgG Alexa Fluor 568 (A11031, 1:1000) and goat anti-rabbit IgG Alexa Fluor 488 (A11034, 1:1000) were purchased from ThermoFisher Scientific. The rabbit anti-puromycin antibody was a gift from Stefan Marciniak (CIMR, Cambridge). Uniformly labelled ^13^C5-glutamine (CLM-1822-H-MPT-PK) was purchased from CK Isotopes. Rotenone, antimycin A, DAPI nitrotetrazolium blue, NADH, aspartate, l-lysine, l-arginine, l-lysine-^13^C_6_, ^15^N_2_ and l-arginine-^13^C_6_, ^15^N_4_ (Arg-10) were purchased from Sigma Aldrich. BAY 87-2243 was purchased from MedChemExpress.

### Gene expression analysis

Total RNA samples were isolated using the GeneElute kit, following the manufacturer’s protocol (Sigma-Aldrich). cDNA synthesis was carried out using the qScript synthesis kit, following the manufacturer’s protocol (Quantabio). mRNA expression was measured by quantitative (Q)-PCR using SYBR Green Mastermix (Eurogentec Ltd.) and the DNA Engine Opticon 2 system (BioRad). Q-PCR primer sequences were as follows: *CHCHD4*_F 5′-GAGCTGAGGAAGGGAAGGAT-3′; *CHCHD4*_R 5′-AATCCATGCTCCTCGTATGG-3′; *KRT18*_F 5′-TAGATGCCCCCAAATCTCAG-3′; *KRT18*_R 5′-CACTGTGGTGCTCTCCTCAA-3′; *CDH2*_F 5′-AGGATCAACCCCATACACCA-3′; *CDH2*_R 5′-TGGTTTGACCACGGTGACTA-3′; *VIM*_F 5′-GAGAACTTTGCCGTTGAAGC-3′; *VIM*_R 5′-TCCAGCAGCTTCCTGTAGGT-3′; *NDI1*_F 5'-AGTCAGATTCGCTTCCACCA-3'; *NDI1*_R 5'-CCCAGTATCAGCACGTTTGG-3';  *ACTB*_F 5′-CCCAGAGCAAGAGAGG-3′; *ACTB*_R 5′-GTCCAGACGCAGGATG-3′

### Mitochondrial fractionation

Crude mitochondrial fractions were prepared from cultured cells as follows. All tubes and reagents were pre-chilled, and all steps were carried out at 4 °C or on ice. Cells were collected and washed twice with homogenisation buffer (HB) (250 mM Mannitol, 5 mM HEPES (pH 7.4), 0.5 mM EGTA, in water). Pellets were resuspended in 1 mL of HB and transferred to a chilled glass potter. Cells were lysed with 150 strokes of potter on ice, and 50 μL of homogenate removed for whole cell lysate (WCL) sample. The remaining lysate was spun at 1000×*g* for 5 min at 4 °C. Supernatants were transferred to fresh tubes and spun at 2000×*g* for 5 min at 4 °C. Supernatants were again transferred to fresh tubes and spun at 10,000×*g* for 10 min at 4 °C. Fifty microlitres of supernatants were retained as the cytoplasm sample. Mitochondrial pellets were washed with 2–3 mL HB and spun at 10,000×*g* for 10 min at 4 °C. Supernatants were carefully removed, and mitochondrial pellets were resuspended in 200 μL HB for functional assays or 200 μL 1× Laemmli sample buffer for immunoblotting.

### Sulforhodamine B viability assay

As previously described [30]. Briefly, cells were plated in appropriate tissue culture vessels, and allowed to adhere overnight prior to treatment. At the end of incubation, media was removed and cells were fixed with 10% trichloroacetic acid (TCA) for 30 min. TCA was washed with water, wells were allowed to air dry and then an excess of 0.4% (*w*/*v*) sulforhodamine B (SRB) in 1% acetic acid was used to stain fixed cells for > 10 min. Excess SRB was washed off with 1% acetic acid solution. Bound SRB was resuspended in a suitable volume of 10 mM Tris, and absorbance of solution measured at 570 nm. For proliferation assays, cells were plated on ‘day − 1’ in triplicate in 12-well plates, and cultured in maintenance DMEM overnight, after which ‘day 0’ plates fixed with TCA. For drug sensitivity assays, cells were plated in triplicate columns in 96-well plates, and cultured in maintenance DMEM overnight. Appropriate wells were dosed with serial dilutions of compounds, including vehicle control wells. Cells were incubated for desired time points followed by SRB assay. To account for any changes in growth rate between cell lines, we also calculated the concentration for growth rate inhibition (GR) at 50% (GR_50_) of the concentration at which maximum growth is inhibited (GRmax) in the presence of drug relative to the untreated control.

### BN-PAGE for CI activity

Samples were prepared for BN-PAGE using the Native-PAGE Sample Preparation Kit and Protocol (Life Technologies) using a 10% dodecylmaltoside (DDM) permeabilization solution. Samples were run on 3–12% gradient non-reducing acrylamide gels (Life Technologies). For complex I activity assay, samples were run without Coomassie blue, and gels incubated in a complex I assay buffer (100 μM NADH and 0.5 mg/mL nitrotetrazolium blue in 20 mM Tris) as previously described [69]. Bovine respiratory chain complex standards were a gift from Judy Hirst (MRC Mitochondrial Biology Unit, Cambridge, UK).

### Microscopy

For immunofluorescence microscopy, cells were seeded onto 13-mm-diameter coverslips, and after treatment, were fixed in ice-cold methanol overnight at − 20 °C. Coverslips were then washed with phosphate-buffered saline (PBS). Immunostaining was carried out by serial incubation using primary antibodies directed to vimentin (rabbit polyclonal) and α-Tubulin (mouse monoclonal), followed by an anti-rabbit Alexa 488 and anti-mouse Alexa 568 secondary antibodies, as well as DAPI (1 μg/mL). All cell imaging was carried out using a DMI4000 B inverted microscope (Leica). Vimentin distribution analysis was carried out using Cell Profiler Image analysis software as we have previously described for analysis of other proteins [29]. Live cell imaging of NADH autofluorescence was carried out according to published protocols [70]. In brief, cells were plated on coverslips and incubated overnight in maintenance DMEM. Coverslips were transferred to custom imaging rings, placed on a heated stage, then 500 μL basal assay media (DMEM supplemented with 25 mM glucose, 1 mM pyruvate, 2 mM glutamax (Thermo Fisher Scientific cat. no. 35050061), 10 mM HEPES, pH 7.4) added above. Intracellular NADH fluorescence intensity time-series were imaged using a Zeiss LSM 510 laser scanning confocal microscope, with sample illumination at 351 nm. Five images were taken, at 1-min intervals under each of the following conditions: (i) basal (untreated), (ii) 1 mM cyanide, (iii) post-wash with fresh medium and (iv) 1 μM FCCP. Image analysis carried out using ImageJ (NIH). Ratio of basal (untreated) NADH pool to total intracellular NAD/NADH pool calculated for each field of view by setting maximal NADH fluorescence (+cyanide) to 100, and minimal NADH fluorescence (+FCCP) to 0. 3 fields of view imaged per cell line. For the scratch wound assay; cells were cultured to 100% confluency, then a single lateral scratch wound was made in each cell monolayer using the point of a disposable pipette tip. Wells were washed twice with PBS to remove cell debris, and then replaced with DMEM containing 10 μg/mL mitomycin C (Sigma Aldrich) for the duration of the assay. Scratch wounds were imaged in three random fields of view using transmitted light and a 10x objective, at both 0 h and after 24 h culture (at 5% CO_2_, 37°C). Scratch wound widths were measured using Leica Application Suite - Advanced Fluorscence software.

### Gene set analysis

Transcriptomic data generated by TCGA was accessed from the CBioPortal data portal (http://www.cbioportal.org/). Samples for gene expression heatmaps were filtered by excluding samples with a z-score value for *CHCHD4* expression > 1.5. Genes for correlation analyses were filtered by excluding samples with a *p* value > 0.05. All correlations were calculated using Spearman’s method. GSEA were carried out via the Broad Institute analysis portal (http://software.broadinstitute.org/gsea/msigdb/index.jsp).

### Western blot densitometry

Western blot signal intensity was measured per lane using ImageJ (NIH) analysis software. Phosphorylated (P)-p70S6K band intensities were normalised to total (T)-p70S6K band intensities, then relative band intensities were calculated compared to untreated control samples for each cell line.

### Metabolomics analysis

For steady-state metabolomics or metabolite tracing experiments, 1 × 10^5^ cells were seeded in 6 wells of a 6-well plate for each cell line. After 24 h, cells were washed twice with PBS and medium was changed with DMEM medium containing metabolite tracers. For glutamine tracing experiments, 6 mM ^13^C5-glutamine was added to glutamine-free DMEM, together with 10% *v*/*v* FBS. After incubation for 24 h with medium containing metabolite tracers, one well was used to estimate cell number. To extract intracellular metabolites, cell plates were placed on ice, washed twice with ice-cold PBS and 1 mL of metabolite extraction buffer (MEB, 50% methanol, 30% acetonitrile, 20% ultrapure water, 100 ng/mL HEPES) per 10^6^ cells was added to each well and cells were scraped. One cycle of freeze-thawing at − 80 °C was performed to further lyse the cells. Intracellular fractions were then incubated in a thermomixer (Eppendorf) at max speed for 15 min at 4 °C. Proteins were then pelleted by centrifuging samples at 16,000×*g* for 10 min at 4 °C and supernatants were transferred into glass vials and stored at − 80 °C until further analysis. Liquid chromatography–mass spectrometry (LC-MS) analysis was performed on a QExactive Orbitrap mass spectrometer coupled to a Dionex UltiMate 3000 Rapid Separation LC system (Thermo). The LC system was fitted with a SeQuant ZIC-pHILIC (150 mm × 2.1 mm, 5 mm) with the corresponding guard column (20 mm × 2.1 mm, 5 mm) both from Merck. The mobile phase was composed of 20 mM ammonium carbonate and 0.1% ammonium hydroxide in water (solvent A), and acetonitrile (solvent B). The flow rate was set at 200 mL/min with a previously described gradient [71]. The mass spectrometer was operated in full MS and polarity switching mode scanning a range of 50–750 *m*/*z*. Samples were randomised, in order to avoid machine drift, and were blinded to the operator. The acquired spectra were analysed using XCalibur Qual Browser and XCalibur Quan Browser software (Thermo Scientific) by referencing to an internal library of compounds. Calibration curves were generated using synthetic standards of the indicated metabolites. Intensity of intracellular metabolites were normalised on total ion sum (normalised intensity values). For interpretation of labelling patterns, normalised intensities of isotopologues were further normalised on total isotopologue sum for each metabolite species (proportion of total pool values).

## Additional file

Additional file 1: **Figure S1.** *CHCHD4* expression positively correlates with OXPHOS and proliferative pathways in tumours. **a** Heatmap of selected genes from HALLMARK_OXIDATIVE_PHOSPHORYLATION gene set (Broad Institute) that are positively correlated with *CHCHD4* expression in Novartis/Broad Institute Cancer Cell Line Encyclopedia RNASeq data. *n* = 967 cell lines. **b** Heatmap of selected genes from HALLMARK_MTORC1 _SIGNALLING gene set (Broad Institute) that are positively correlated with *CHCHD4* expression in Novartis/Broad Institute Cancer Cell Line Encyclopedia RNASeq data. *n* = 967 cell lines. **c** Chart shows GSEA of genes positively correlated with *CHCHD4* expression in glioblastoma patient tumours. (PDF 153 kb)

Additional file 2: **Figure S2.** CHCHD4-mediated tumour cell growth and mTORC1 signalling is coupled to CI activity. **a** Western blots show levels of AIF, and myc-tagged CHCHD4 in control U2OS cells, and cells overexpressing wild-type (WT.cl1, WT.cl3) or mutant (C66A/C68A) CHCHD4. β-Actin was used as a load control. **b** Chart shows mean fluorescence intensity of NADH in control U2OS cells, and U2OS cells expressing exogenous CHCHD4 (WT.cl1). Cells treated at indicated time points with 1 mM cyanide (CN) and 1 µM FCCP. 1 image per minute, 5 images per treatment, 3 fields of view per cell line. ±SD. *n* = 3. Representative images of control U2OS cells at each condition also shown. **c** Western blots show levels of phosphorylated (P-) and total (T-) p70S6K, and puromycin labelled polypeptides in control U2OS cells treated with 0, 50 or 100 nM rotenone for 24 h, in the absence (NT) or presence of 10 mM aspartate (+D). β-Actin was used as a load control. **d** Agarose gel shows expression of *NDI1* transcript in control U2OS cells and cells expressing CHCHD4 (WT.cl1), stably transfected with empty vector (pWPI) or NDI1-containing vector (NDI1). *ACTB* transcript expression was used as a control. (PDF 273 kb)

Additional file 3: **Figure S3.** CHCHD4 expression links growth rate to CI activity, and correlates with tumour cell doubling time. **a** Chart shows growth of tumour cell line panel treated with 500 nM BAY 87-2243 for 72 h, relative to untreated (0 nM) cells. ±SD. *n* = 3. **b** Chart shows growth of tumour cell line panel treated with 3 µM antimycin A for 72 h, relative to untreated (0 nM) cells. ±SD. *n* = 3. **c** Chart shows xy scatter of CHCHD4 transcript levels (RPKM - Reads Per Kilobase of transcript per Million mapped reads), and doubling times for 368 tumour cell lines. Trend line (dashed black), R^2^ value (Spearman’s correlation) and *p*-value of correlation shown. (PDF 110 kb)

Additional file 4: **Figure S4.** CHCHD4-mediated tumour cell growth is linked to CI-regulated mTORC1 signalling and amino acid metabolism. Chart shows extracellular levels of glutamine measured in culture medium from control U2OS cells, and cells expressing wild-type CHCHD4 (WT.cl1). Representative of 2 experiments. ±SD. *n* = 5. (PDF 61 kb)

Additional file 5: **Figure S5.** CHCHD4 regulates the EMT phenotype of tumour cells. **a-b** Charts show GSEA of genes negatively correlated with *CHCHD4* expression in (**a**) breast cancer and (**b**) colon adenocarcinoma patient tumours. **c** Chart shows GSEA of genes negatively correlated with *CHCHD4* expression in Novartis/Broad Institute Cell Line Encyclopedia. *n* = 967 cell lines. **d** Chart shows densitometry analysis of vimentin band intensity from 3 independent western blots as described in Fig. 5c. ±SD. *n* = 3. **e** Western blots show levels of E-cadherin and myc-tagged CHCHD4 in control (Ctrl) HCT116 cells, and cells overexpressing wild-type CHCHD4 (WT.cl8). β-Actin was used as a load control. **f** Western blots show levels of E-cadherin and CHCHD4 in HCT116 cells stably expressing control (Ctrl) shRNA or shRNA targeting CHCHD4 (CHCHD4 shRNA). β-Actin was used as a load control. **g** Chart shows relative proportion of fluorescently labelled vimentin in the perinuclear and peripheral sections of control U2OS cells and cells overexpressing wild-type CHCHD4 (WT.cl1) untreated (NT) or treated with 50 nM rotenone for 72 h. ±SD. *n* = 2 experiments, 5 fields of view per condition. (PDF 175 kb)
