# Supplementary material for: CHCHD4 regulates tumour proliferation and EMT-related phenotypes, through respiratory chain-mediated metabolism
Source: Cancer Metab. 2019 Jul 16;7:7. doi: 10.1186/s40170-019-0200-4 (PMC6632184; doi:10.1186/s40170-019-0200-4)
Supplement: Supplementary file 1 — Figure S1. CHCHD4 expression positively correlates with OXPHOS and proliferative pathways in tumours. a Heatmap of selected genes from HALLMARK_OXIDATIVE_PHOSPHORYLATION gene set (Broad Institute) that are positively correlated with CHCHD4 expression in Novartis/Broad Institute Cancer Cell Line Encyclopedia RNASeq data. n = 967 cell lines. b Heatmap of selected genes from HALLMARK_MTORC1 _SIGNALLING gene set (Broad Institute) that are positively correlated with CHCHD4 expression in Novartis/Broad Institute Cancer Cell Line Encyclopedia RNASeq data. n = 967 cell lines. c Chart shows GSEA of genes positively correlated with CHCHD4 expression in glioblastoma patient tumours. (PDF 153 kb) [file 40170_2019_200_MOESM1_ESM.pdf]

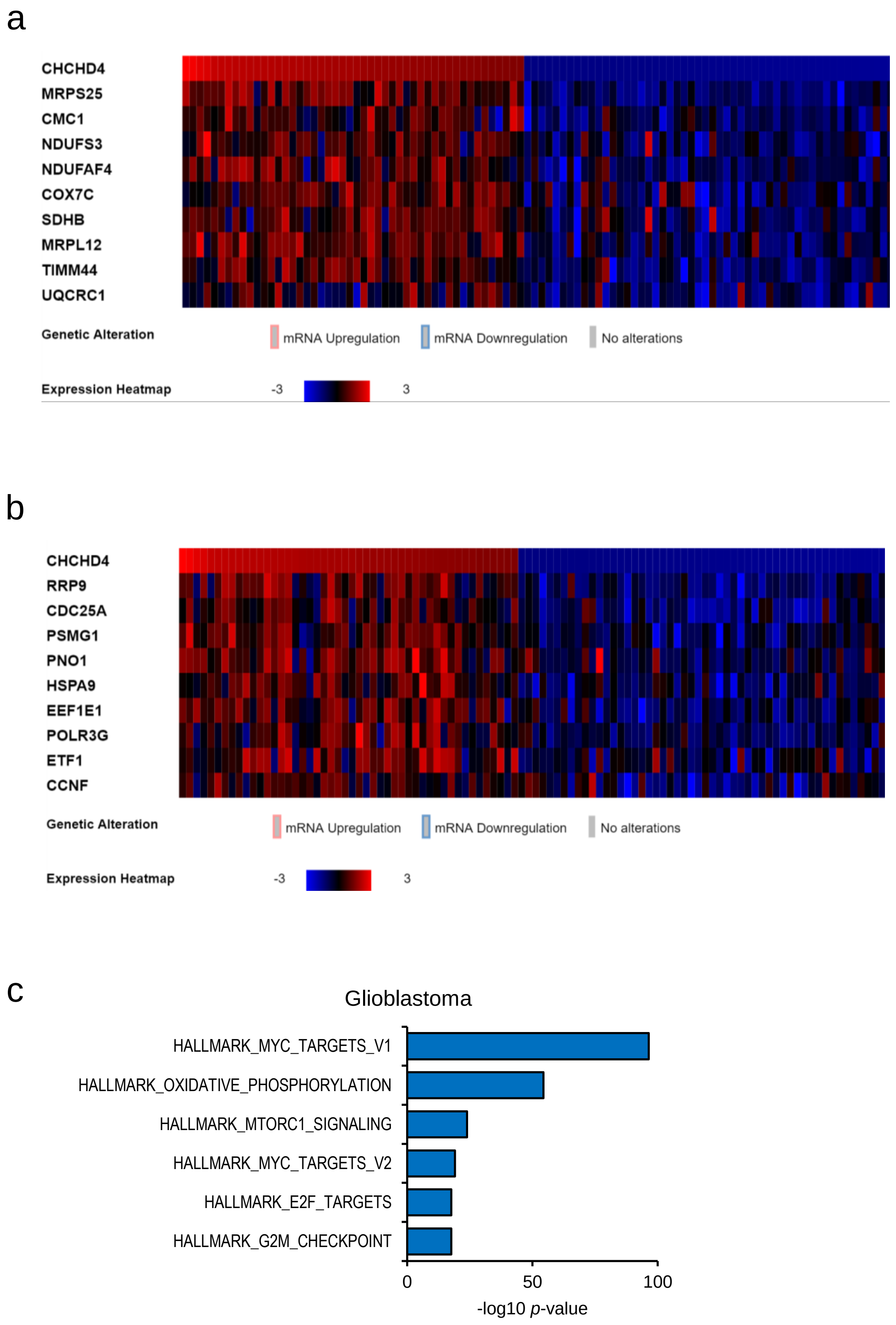

Additional File 1. Figure S1. ***CHCHD4*** **expression positively correlates with OXPHOS and proliferative pathways in tumours**

Figure S1. CHCHD4 expression positively correlates with OXPHOS and proliferative pathways in tumours. **a** Heatmap of selected genes from HALLMARK_OXIDATIVE_PHOSPHORYLATION gene set (Broad Institute) that are positively correlated with *CHCHD4* expression in Novartis/Broad Institute Cancer Cell Line Encyclopedia RNASeq data. *n* = 967 cell lines. **b** Heatmap of selected genes from HALLMARK_MTORC1 _SIGNALLING gene set (Broad Institute) that are positively correlated with *CHCHD4* expression in Novartis/Broad Institute Cancer Cell Line Encyclopedia RNASeq data. *n* = 967 cell lines. **c** Chart shows GSEA of genes positively correlated with *CHCHD4* expression in glioblastoma patient tumours.
