# Supplementary material for: CHCHD4 regulates tumour proliferation and EMT-related phenotypes, through respiratory chain-mediated metabolism
Source: Cancer Metab. 2019 Jul 16;7:7. doi: 10.1186/s40170-019-0200-4 (PMC6632184; doi:10.1186/s40170-019-0200-4)
Supplement: Supplementary file 2 — Figure S2. CHCHD4-mediated tumour cell growth and mTORC1 signalling is coupled to CI activity. a Western blots show levels of AIF, and myc-tagged CHCHD4 in control U2OS cells, and cells overexpressing wild-type (WT.cl1, WT.cl3) or mutant (C66A/C68A) CHCHD4. β-Actin was used as a load control. b Chart shows mean fluorescence intensity of NADH in control U2OS cells, and U2OS cells expressing exogenous CHCHD4 (WT.cl1). Cells treated at indicated time points with 1 mM cyanide (CN) and 1 µM FCCP. 1 image per minute, 5 images per treatment, 3 fields of view per cell line. ±SD. n = 3. Representative images of control U2OS cells at each condition also shown. c Western blots show levels of phosphorylated (P-) and total (T-) p70S6K, and puromycin labelled polypeptides in control U2OS cells treated with 0, 50 or 100 nM rotenone for 24 h, in the absence (NT) or presence of 10 mM aspartate (+D). β-Actin was used as a load control. d Agarose gel shows expression of NDI1 transcript in control U2OS cells and cells expressing CHCHD4 (WT.cl1), stably transfected with empty vector (pWPI) or NDI1-containing vector (NDI1). ACTB transcript expression was used as a control. (PDF 273 kb) [file 40170_2019_200_MOESM2_ESM.pdf]

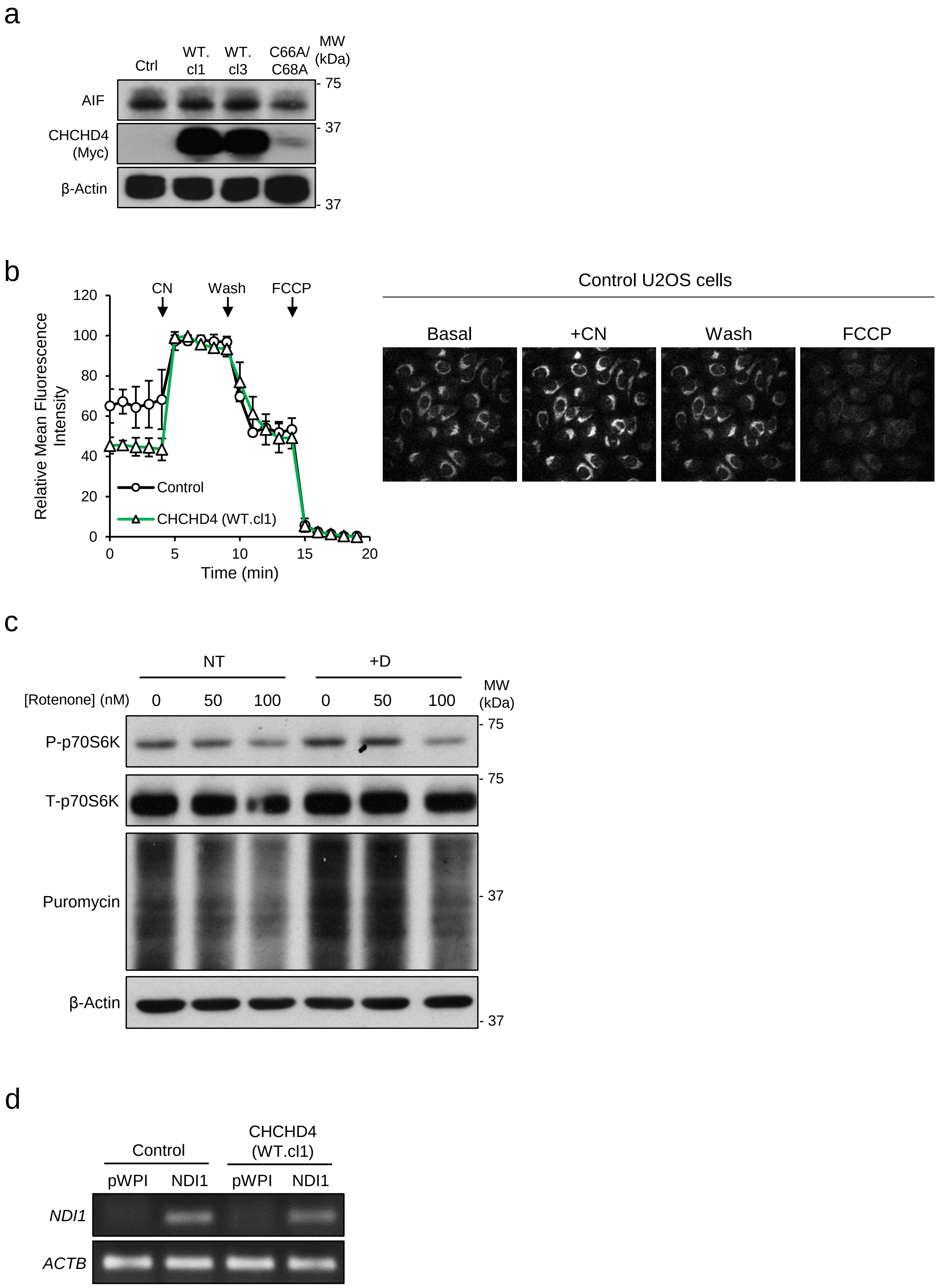

Additional File 2: Figure S2. CHCHD4-mediated tumour cell growth and mTORC1 signalling is coupled to complex I activity.

Figure S2. CHCHD4-mediated tumour cell growth and mTORC1 signalling is coupled to CI activity. **a** Western blots show levels of AIF, and myc-tagged CHCHD4 in control U2OS cells, and cells overexpressing wild-type (WT.cl1, WT.cl3) or mutant (C66A/C68A) CHCHD4. β-Actin was used as a load control. **b** Chart shows mean fluorescence intensity of NADH in control U2OS cells, and U2OS cells expressing exogenous CHCHD4 (WT.cl1). Cells treated at indicated time points with 1 mM cyanide (CN) and 1 µM FCCP. 1 image per minute, 5 images per treatment, 3 fields of view per cell line. ±SD. n = 3. Representative images of control U2OS cells at each condition also shown. **c** Western blots show levels of phosphorylated (P-) and total (T-) p70S6K, and puromycin labelled polypeptides in control U2OS cells treated with 0, 50 or 100 nM rotenone for 24 h, in the absence (NT) or presence of 10 mM aspartate (+D). β-Actin was used as a load control. **d** Agarose gel shows expression of *NDI1* transcript in control U2OS cells and cells expressing CHCHD4 (WT.cl1), stably transfected with empty vector (pWPI) or NDI1-containing vector (NDI1). *ACTB* transcript expression was used as a control.
