# Supplementary material for: CHCHD4 regulates tumour proliferation and EMT-related phenotypes, through respiratory chain-mediated metabolism
Source: Cancer Metab. 2019 Jul 16;7:7. doi: 10.1186/s40170-019-0200-4 (PMC6632184; doi:10.1186/s40170-019-0200-4)
Supplement: Supplementary file 3 — Figure S3. CHCHD4 expression links growth rate to CI activity, and correlates with tumour cell doubling time. a Chart shows growth of tumour cell line panel treated with 500 nM BAY 87-2243 for 72 h, relative to untreated (0 nM) cells. ±SD. n = 3. b Chart shows growth of tumour cell line panel treated with 3 µM antimycin A for 72 h, relative to untreated (0 nM) cells. ±SD. n = 3. c Chart shows xy scatter of CHCHD4 transcript levels (RPKM - Reads Per Kilobase of transcript per Million mapped reads), and doubling times for 368 tumour cell lines. Trend line (dashed black), R2 value (Spearman’s correlation) and p-value of correlation shown. (PDF 110 kb) [file 40170_2019_200_MOESM3_ESM.pdf]

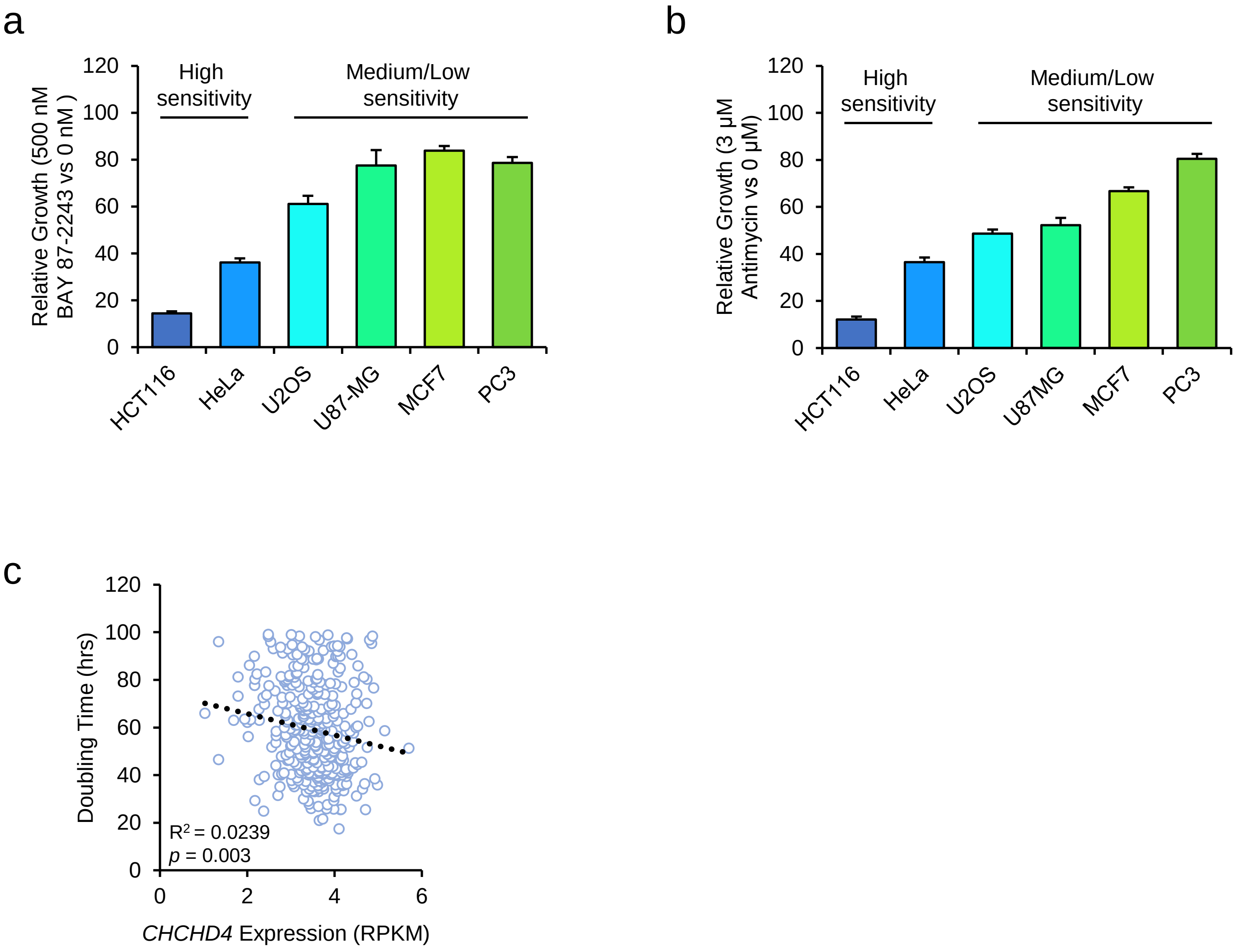

Additional File 3: Figure S3. CHCHD4 expression links growth rate to CI activity, and correlates with tumour cell doubling time.

Figure S3. CHCHD4 expression links growth rate to CI activity, and correlates with tumour cell doubling time. **a** Chart shows growth of tumour cell line panel treated with 500 nM BAY 87-2243 for 72 h, relative to untreated (0 nM) cells. ±SD. $n = 3$. **b** Chart shows growth of tumour cell line panel treated with 3 µM antimycin A for 72 h, relative to untreated (0 nM) cells. ±SD. $n = 3$. **c** Chart shows xy scatter of *CHCHD4* transcript levels (RPKM - Reads Per Kilobase of transcript per Million mapped reads), and doubling times for 368 tumour cell lines. Trend line (dashed black), R^2 value (Spearman's correlation) and *p*-value of correlation shown.
