# Supplementary material for: CHCHD4 regulates tumour proliferation and EMT-related phenotypes, through respiratory chain-mediated metabolism
Source: Cancer Metab. 2019 Jul 16;7:7. doi: 10.1186/s40170-019-0200-4 (PMC6632184; doi:10.1186/s40170-019-0200-4)
Supplement: Supplementary file 4 — Figure S4. CHCHD4-mediated tumour cell growth is linked to CI-regulated mTORC1 signalling and amino acid metabolism. Chart shows extracellular levels of glutamine measured in culture medium from control U2OS cells, and cells expressing wild-type CHCHD4 (WT.cl1). Representative of 2 experiments. ±SD. n = 5. (PDF 61 kb) [file 40170_2019_200_MOESM4_ESM.pdf]

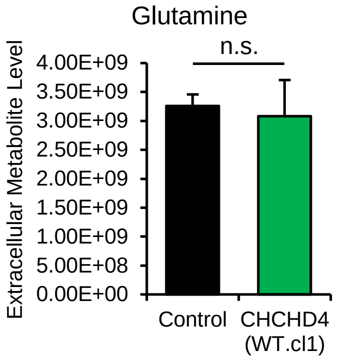

Additional File 4: Figure S4. CHCHD4-mediated tumour cell growth is linked to CI-regulated mTORC1 signalling and amino acid metabolism.

Figure S4. CHCHD4-mediated tumour cell growth is linked to CI-regulated mTORC1 signalling and amino acid metabolism. Chart shows extracellular levels of glutamine measured in culture medium from control U2OS cells, and cells expressing wild-type CHCHD4 (WT.cl1). Representative of 2 experiments. ±SD. $n = 5$.
