# Supplementary material for: CHCHD4 regulates tumour proliferation and EMT-related phenotypes, through respiratory chain-mediated metabolism
Source: Cancer Metab. 2019 Jul 16;7:7. doi: 10.1186/s40170-019-0200-4 (PMC6632184; doi:10.1186/s40170-019-0200-4)
Supplement: Supplementary file 5 — Figure S5. CHCHD4 regulates the EMT phenotype of tumour cells. a-b Charts show GSEA of genes negatively correlated with CHCHD4 expression in (a) breast cancer and (b) colon adenocarcinoma patient tumours. c Chart shows GSEA of genes negatively correlated with CHCHD4 expression in Novartis/Broad Institute Cell Line Encyclopedia. n = 967 cell lines. d Chart shows densitometry analysis of vimentin band intensity from 3 independent western blots as described in Fig. 5c. ±SD. n = 3. e Western blots show levels of E-cadherin and myc-tagged CHCHD4 in control (Ctrl) HCT116 cells, and cells overexpressing wild-type CHCHD4 (WT.cl8). β-Actin was used as a load control. f Western blots show levels of E-cadherin and CHCHD4 in HCT116 cells stably expressing control (Ctrl) shRNA or shRNA targeting CHCHD4 (CHCHD4 shRNA). β-Actin was used as a load control. g Chart shows relative proportion of fluorescently labelled vimentin in the perinuclear and peripheral sections of control U2OS cells and cells overexpressing wild-type CHCHD4 (WT.cl1) untreated (NT) or treated with 50 nM rotenone for 72 h. ±SD. n = 2 experiments, 5 fields of view per condition. (PDF 175 kb) [file 40170_2019_200_MOESM5_ESM.pdf]

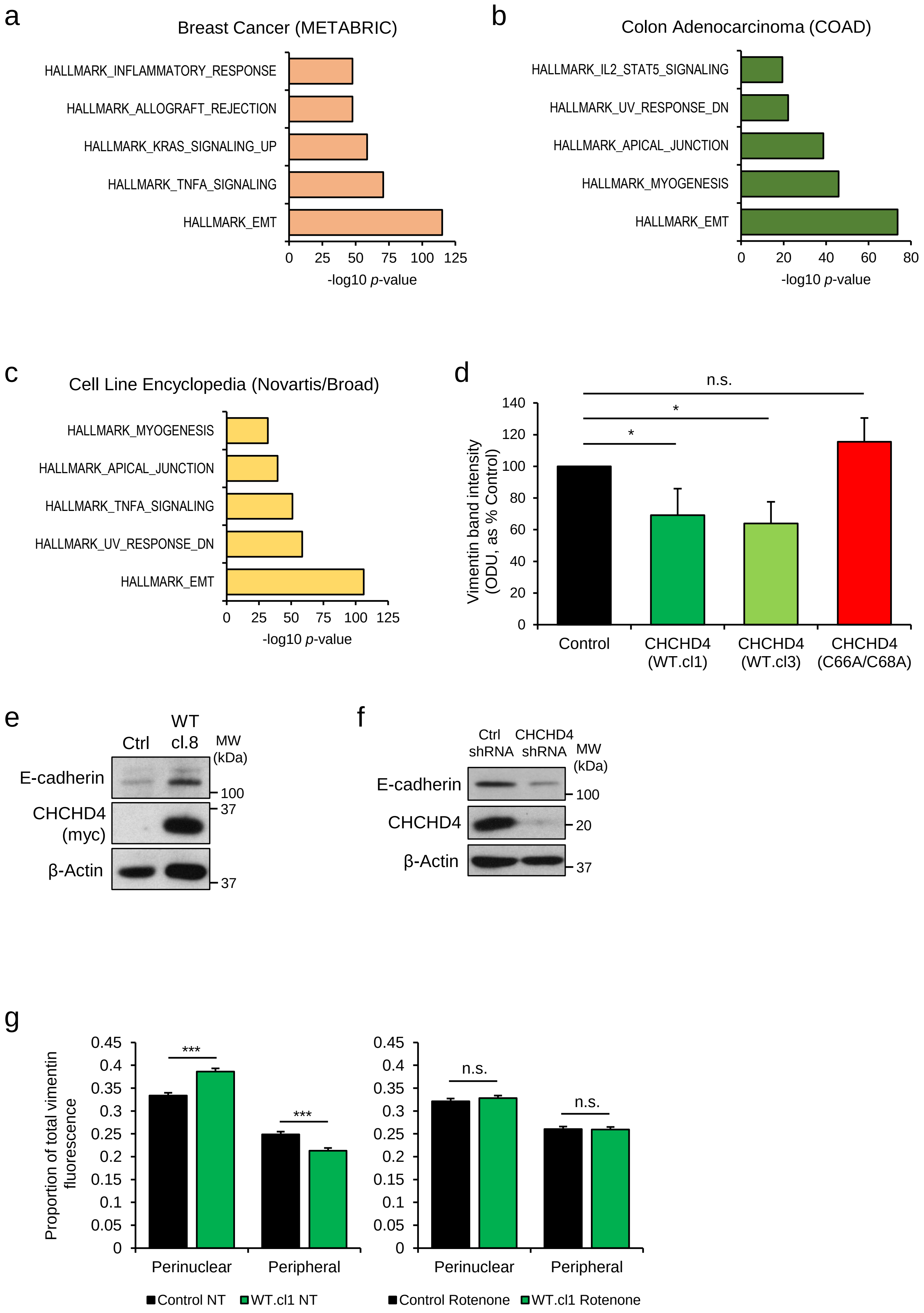

Additional File 5: Figure S5. CHCHD4 regulates the EMT phenotype of tumour cells.

Figure S5. CHCHD4 regulates the EMT phenotype of tumour cells. **a-b** Charts show GSEA of genes negatively correlated with *CHCHD4* expression in (**a**) breast cancer and (**b**) colon adenocarcinoma patient tumours. **c** Chart shows GSEA of genes negatively correlated with *CHCHD4* expression in Novartis/Broad Institute Cell Line Encyclopedia. n = 967 cell lines. **d** Chart shows densitometry analysis of vimentin band intensity from 3 independent western blots as described in Fig. 5c. ±SD. n = 3. **e** Western blots show levels of E-cadherin and myc-tagged CHCHD4 in control (Ctrl) HCT116 cells, and cells overexpressing wild-type CHCHD4 (WT.cl8). β-Actin was used as a load control. **f** Western blots show levels of E-cadherin and CHCHD4 in HCT116 cells stably expressing control (Ctrl) shRNA or shRNA targeting CHCHD4 (CHCHD4 shRNA). β-Actin was used as a load control. **g** Chart shows relative proportion of fluorescently labelled vimentin in the perinuclear and peripheral sections of control U2OS cells and cells overexpressing wild-type CHCHD4 (WT.cl1) untreated (NT) or treated with 50 nM rotenone for 72 h. ±SD. n = 2 experiments, 5 fields of view per condition.
